# Public health intelligence challenges for local public health authorities responding to disease outbreaks: a mixed-methods systematic review protocol

**DOI:** 10.3310/nihropenres.13307.2

**Published:** 2023-07-19

**Authors:** Janette Parr, Yen-Fu Chen, Sarah Damery, Amy Grove

**Affiliations:** 1 Warwick Medical School, The University of Warwick, Coventry, West Midlands, CV4 7AL, UK; 2 Institute of Applied Health Research, University of Birmingham, Birmingham, West Midlands, B15 2TT, UK

**Keywords:** Public Health Systems Research, Public Health, Public Health Intelligence, Public Health Informatics, Disease Outbreaks, COVID-19, Public Health Authority, Local Government

## Abstract

**Background:**

Information management capacity is crucial for controlling risks from health emergencies. But little is known about how sub-national public health bodies overcome public health intelligence challenges when responding to disease outbreaks. This paper describes a protocol for a mixed-methods systematic review to fill this knowledge gap. In addition to describing the evidence base and characterising public health intelligence responses, it will explore reported facilitators and barriers to response.

**Methods:**

Research on sub-national Public Health Intelligence disease outbreak responses will be synthesised. The review will be limited to articles published in English, during or after 2019. Key electronic databases will be searched for peer-reviewed, primary research studies. Two reviewers will independently screen articles for relevance. Articles that refer to a public health intelligence response to a propagated disease outbreak by a sub-national Public Health Authority will be included. Quality assessment of included articles will be undertaken using published tools. Data integration will be by the Pillar Integration Process (PIP).

**Discussion:**

This review will describe and synthesise the recent literature on sub-national Public Health Authorities’ responses to propagated disease outbreaks. The systematic design will limit bias and the inclusion of data from quantitative, qualitative and mixed-methods studies will ensure relevant evidence is considered regardless of the methodology used to produce it. The review is part of a larger research project which aims to explore the role of sub-national public health intelligence during the COVID-19 pandemic and investigate how public health intelligence preparedness could be improved in the future. This could provide information to support the development of training, preparedness indicators and/or ways of implementing directives.

**PROSPERO registration:**

CRD42022308042 (08/02/2022)

## Plain English summary

What are the information challenges for local public health services responding to an outbreak of disease?

Useful information is vital for planning public health activities. This means good information management is very important during an outbreak of disease. But little is known about how local public health bodies overcome challenges in this area. Examples of challenges could include not receiving sufficient data and/or not having enough staff to analyse it.

This paper describes planned research based on a review of the literature. We want to know how local public health bodies have collected, analysed and provided useful information during disease outbreaks and what makes it easier or harder for them to do this. To make the results more reliable, the review will take a structured approach. Two people will work on some stages to check each other’s work.

We will search databases of research articles to find any kind of study that describes information challenges for local public health organisations during a disease outbreak. Specific criteria will be used to judge which studies to include. To be included, studies must be about a disease that spreads from person-to-person, and they must have been published in or after 2019. Included studies will be summarised.

Results will feed into further research on how local public health bodies could be better prepared for disease outbreaks in the future.

## Introduction

Worldwide, many types of hazardous events, including infectious disease outbreaks, are increasing in frequency ( World Health Organization, 2019). Without effective risk management, these events may lead to emergencies and disasters which in turn may have devastating health, economic, political, and societal consequences. To help stakeholders address these risks the World Health Organization (WHO) has developed the Health Emergency and Disaster Risk Management Framework (Health EDRM) ( World Health Organization, 2019). The Health EDRM is a conceptual framework intended to consolidate practice and assist all parties in adopting an approach which prioritises preparation. Information and knowledge management is a defined component of the Health EDRM. The WHO advocate planning for staffing and training at all levels of this function ( World Health Organization, 2019).

In England, many public health services including some information and knowledge management functions are delivered by local governments (also known as local authorities). However, a report from the House of Commons Health and Social Care, and Science and Technology Committees on the United Kingdoms’ response to the coronavirus disease 2019 (COVID-19) pandemic describes a perceived failure to value public health at the local level ( House of Commons, 2021). Indeed, The Local Government Association has stated that had the role of local public health been clearly recognised, measures such as contact tracing would have been rolled out quicker ( Local Government Association, 2021). They have also highlighted poor understanding of roles and responsibilities, levers, and powers as key fault lines in the system ( Cross Party Local Government Association, 2021). In England emergency preparedness, response and recovery is co-ordinated by multi-agency partnerships which incorporate local authorities. These partnerships are called Local Resilience Forums (LRFs). Importantly, LRFs are not separately resourced meaning they have no access to capacity beyond individual partners ( Local Government Association, no date).

Even before the COVID-19 pandemic, there were documented concerns around the public health information and knowledge workforce. These included the effects of team reorganisations, funding reduction, lack of clear career pathways, workforce immobility and insufficient support from national bodies ( Centre for Workforce Intelligence, 2015 and Shickle *et al*., 2018). Understanding levels of preparedness in advance of a health emergency, has been highlighted as an important knowledge gap by the WHO ( World Health Organization, 2021). This research will explore the challenges sub-national Public Health Authorities have experienced in responding to the COVID-19 pandemic as part of a wider project to explore the English experience.

A preliminary literature search identified no existing or ongoing systematic reviews on sub-national information and knowledge management challenges during disease outbreaks. A recent narrative review, global in scope, describes the main health information management challenges during COVID-19 as:

(1) lack of standards for information exchange between Clinical Healthcare Providers and Public Health Authorities
(2) problems in data collection and data quality, especially in terms of completeness and timeliness
(3) governance, public policies, and regulations

( Massoudi and Sobolevskaia, 2021)

However, Massoudi and Sobolevskaia do not provide detail on the challenges sub-national Public Health Authorities have faced. And although the authors highlight workforce issues as important, the review does not directly address these. It is pertinent to examine workforce issues because the WHO have stated the public health workforce’s role is often overlooked during implementation of international regulations to improve health security ( World Health Organization, 2022).

**Figure 1.**
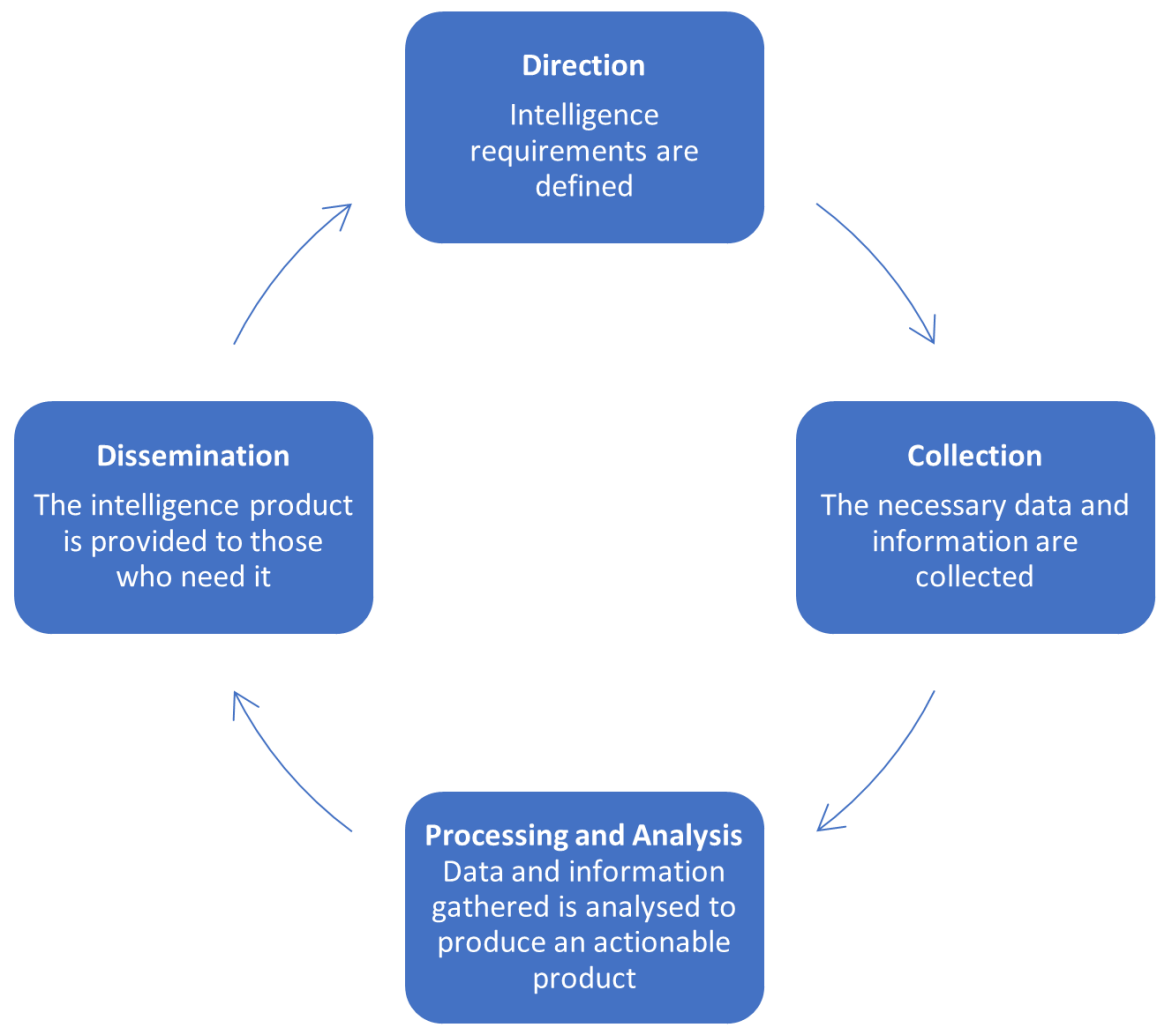
A conceptualisation of the Intelligence Cycle described by Bowsher *et al.*, 2020.

In the UK, information and knowledge management in public health is also called Public Health Intelligence. The concept of Public Health Intelligence, as a defined public health discipline, has gradually emerged and developed in complexity. Public Health Intelligence can be portrayed as spanning the full intelligence cycle ( Regmi & Gee, 2016) which Bowsher *et al*., 2020 describe as encompassing direction, collection, processing and analysis and dissemination ( Figure 1).

The cycle can be understood as a process that transforms data into public health intelligence. Here, intelligence is distinct from data, because it enables evidence-based decisions which are actionable. In other words - intelligence becomes information that is **useful**. Assessing health needs is the starting point for public health activities and therefore, an information system is a core aspect of any public health activity ( Regmi & Gee, 2016).

This paper is the protocol for a mixed-methods systematic review which will inform a larger mixed-methods research project. The larger project will explore the challenges that English local authorities have experienced in responding to the COVID-19 pandemic from a public health intelligence perspective. The review will fill knowledge gaps by synthesising literature on sub-national public health intelligence responses to infectious disease outbreaks. Facilitators and barriers to a response and how preparedness for disease outbreaks could be improved will be explored. Emphasis on workforce issues will allow the examination of the contribution of workforce planning and training as advocated by WHO ( World Health Organization, 2019).

The review is not limited to the English public health system or to just COVID-19 outbreaks. This is so comparisons can be made across countries and types of outbreak. The results will elucidate the facilitators and barriers to sub-national public health intelligence responses.

## Research questions

In relation to propagated disease outbreaks, key questions the review will address are:

- What public health intelligence activities have been undertaken by subnational public health authorities during disease outbreaks, what knowledge, skills and tools were used and how does this differ to routine activity, knowledge, skills, and tools?
- How were local public health intelligence responses organised and resourced (structured) and how did they evolve over time?
- What are the barriers and facilitators to local public health intelligence responses at a personal, team, organisational and system level?
- What should be done to maintain or improve local public health intelligence preparedness for future disease outbreaks?

## Methods

A protocol has been registered with the PROSPERO International Prospective Register of Systematic Reviews on 8 ^th^ February 2022. The registration number is CRD42022308042.

To minimise bias, the review aims to identify, evaluate, summarise, and synthesise studies in a systematic way. Quantitative, qualitative and mixed-methods studies may all address the research questions therefore a mixed-methods convergent integrated approach is appropriate for synthesis and integration ( Lizarondo *et al.*, 2020).

### Data sources and search

To identify relevant articles the following electronic databases will be searched:

• PubMed

• Embase

• Applied Social Sciences Indexes and Abstracts (ASSIA)

• Scopus

• Health Management Information Consortium (HMIC)

• WHO Global Health Library

• Health Systems Evidence

• PDQ Evidence

Searching will be supplemented by:

•   Searching reference lists of included articles

Search strategies for the PubMed and Embase databases are available as *Extended data* ( Parr, 2022). Please see the Data availability section for details.

### Study eligibility

The review questions were developed using the Setting, Perspective, Intervention, Comparison and Evaluation (SPICE) framework as described by Booth, 2006. Table 1 shows the inclusion criteria and how they relate to different elements of the SPICE framework.

**Table 1.** Inclusion criteria.

Framework element	Detail - inclusion criteria underlined/in bold
Setting	**A sub-national Public Health Authority** anywhere in the world. A public health authority is defined as an organisation that provides public health services
Perspective	Staff involved in Public Health Intelligence activities during a propagated disease outbreak
Intervention/exposure of interest	**Public Health Intelligence activities during a propagated disease outbreak** Public Health Intelligence activities are defined as direction, collection, processing and analysis, and dissemination of public health data and information, including research evidence. A propagated disease outbreak is defined as a sudden increase in the incidence of a disease which spreads person-to-person ( CDC, 2012 and National Library of Medicine, 2022). Studies that relate only to insect borne diseases, animal diseases and non-infectious diseases will be excluded.
Comparison	Public Health Intelligence activities when there is no propagated disease outbreak
Evaluation	Outcomes as reported in the literature Stakeholder experiences and perceptions including facilitators and barriers to response and what can be done to improve preparedness Activities performed, skills and tools used to undertake these activities Structures (e.g. organisation and resourcing) used to deliver the response and their evolution over time
Study design	**Peer-reviewed, primary research studies of all designs** including quantitative, qualitative and mixed- methods Non-peer reviewed articles such as theses, reports and conference abstracts and non-empirical articles such as editorials and commentaries will be excluded

Peer-reviewed, primary research studies of all designs will be eligible for inclusion in the review. To be included they must relate to public health intelligence activities by a sub-national public health authority during a propagated disease outbreak. Non-peer-reviewed articles such as theses, reports and conference abstracts and non-research articles such as editorials and commentaries will be excluded. Only articles published in English, in or after January 2019, will be included. The date limit will ensure that information is up to date and that responses to the COVID-19 pandemic are captured.

### Study selection

Following the search for articles, citations will be imported into EndNote and duplicates will be removed. The remaining references will then be exported to the Rayyan webtool for the screening and selection of studies.

Titles and abstracts will be screened independently by two reviewers (JP, SD) using the inclusion criteria listed in Table 1. If no abstract is available, or if it is not possible to determine from the title and abstract whether the article meets inclusion criteria, the full text will be obtained. If disagreements arise, these will be resolved through discussion between reviewers. If after discussion there is still disagreement, conflicts will be resolved by consultation with a third reviewer.

The full text of those articles that meet the initial screening criteria will be obtained and screened using the eligibility criteria. If disagreements arise, these will be resolved through discussion, or arbitration with a third reviewer. Finally, the full texts of all relevant studies found to meet the inclusion criteria will be retained for data extraction and synthesis. Backward citation searches of key articles will be conducted to identify additional studies which may be of interest.

### Data extraction

Data will be extracted from eligible full-text papers using a data extraction form. The form is available as *Extended data* ( Parr, 2022). Please see the Data availability section for details. The form will be piloted by reviewers with a selection of studies of different designs, qualitative, quantitative, and mixed methods, to ensure it is suitable for all.

Descriptive data from a sample of 10% of papers will be extracted by a single reviewer and then cross-checked by a second reviewer. When accuracy is agreed, the remaining papers will be extracted and analysed by a single reviewer.

Different countries may have non-comparable health/public health systems. Therefore, the United Nations Development Programme (UNDP) Human Development Index will be used to classify settings and an analysis of similarities and differences in results between countries of different classifications will be made ( United Nations Development Programme, 2022).

### Quality assessment

Although quality assessment will not be used to exclude studies from the analysis, an assessment of quality will be made available for each article as advocated by Mays *et al*. (2005). All mixed-methods and quantitative studies will be quality assessed using the Mixed Methods Appraisal Tool (MMAT) described by Hong *et al*. (2019). Qualitative studies will be assessed using the JBI Checklist for Qualitative Research ( Lockwood *et al*., 2015). A sample of 10% of the papers will be independently quality assessed by two reviewers. If there is good agreement, the remaining papers will be quality assessed by a single reviewer. Results of the quality assessment will be presented in the final appendix and summary results described narratively in the final manuscript.

### Analysis and integration

Data will be analysed using NVivo software. Thematic Synthesis as described by Langlois *et al*. (2018) will be used to analyse relevant textual data. This will involve a three-step process: 1) line by line coding of the text, 2) development of descriptive themes, 3) development of analytic themes. Quantitative data will be analysed separately to qualitative data. For quantitative data, verbatim textual descriptions of findings will be coded instead of raw numerical data. The Pillar Integration Process (PIP), as described by Johnson *et al*. (2019), will then be used to integrate findings. PIP is a four-stage technique that merges qualitative and quantitative findings into a joint display. The stages are completed sequentially and include: listing, matching, checking, and pillar building.

Robustness of themes will be checked with the author team and by presentation to a Patient and Public Involvement (PPI) group within the Applied Research Collaboration (ARC) West Midlands.

The findings will include:

- A description of the evidence base including the quality of included studies and evidence gaps. This will include whether studies measured response effectiveness and, if so, how they went about this.
- Characterisation of reported local public health intelligence responses to propagated disease outbreaks (including resourcing, organisation and activities)
- Themes around reported facilitators and barriers to response and how preparation could be improved
- Consistency/inconsistency between settings including an assessment using the Human Development Index classification

## Discussion

This research will systematically review recent studies that relate to sub-national public health authorities’ public health intelligence responses during disease outbreaks. The review will identify and synthesise the facilitators and barriers to an outbreak response identified in the primary literature. This will fill important knowledge gaps regarding sub-national responses and workforce-related issues. The findings will provide information to support the organisation of services, training development, preparedness assessment and/or implementation of directives.

Whilst this study is not specifically designed to compare how public health intelligence activities which emerged in 2020 (the pandemic period), differed to pre-pandemic activities or how the emergence of social media effected responses, these comparisons are inherently covered by the research questions.

The focus on sub-national public health entities is intended to allow exploration of facilitators and barriers to disease outbreak response at a local level. This enhanced focus will allow operational aspects, including workforce issues, to be explored in detail. Indeed, previous work has highlighted knowledge gaps around public health preparedness indicators at the local and regional level ( Ontario Agency for Health Protection and Promotion/Public Health Ontario, 2020).

Lee *et al*. (2023) conducted a scoping review exploring priority areas and indicators for Public Health Emergency Preparedness (PHEP) with a focus on infectious disease emergencies. The review identified several ‘emerging’ themes. These included: planning to mitigate inequities, research and evidence-informed decision making, building vaccination capacity, building laboratory and diagnostic system capacity, building infection prevention and control capacity, financial investment in infrastructure, health system capacity, climate and environmental health, public health legislation and phases of preparedness.

This research will contribute to a better response to public health emergencies as the findings can be compared to existing work on local indicators of PHEP, including that undertaken by the Ontario Agency for Health Protection and Promotion (2020) and Lee *et al*., 2023. In relation to public health intelligence, the review will provide empirically derived support for previously identified indicators, explore ‘emerging’ themes, and identify potential new indicators of preparedness.

Other anticipated findings include: 1) a description of the evidence base, 2) reported response characteristics (what was done and how this was organised and resourced) and 3) a summary of recommendations. Findings will be presented by considering individual, team, organisational and system levels, where appropriate, as identified during analysis. Whilst the results will be written up as part of a PhD thesis and shared at relevant seminars and symposiums, a publication in an academic journal is also planned.

### Study status

At the time of writing, article screening has been completed and data extraction is in progress.

## Strengths and limitations

The rigorous mixed-methods design will limit bias and ensure appropriate evidence is considered regardless of its research methodology i.e., whether it is from a quantitative, qualitative or mixed-methods study.

## Amendments

The PROSPERO record was revised after piloting of the inclusion criteria revealed a very large number of studies would need to be full text screened. A decision was taken to limit the review to peer reviewed literature and to apply a date limit.

## Data Availability

No underlying data are associated with this article. Open Science Framework: Public Health Intelligence Challenges for Sub-national Public Health Authorities Responding to Disease Outbreaks: A Mixed-Methods Systematic Review Protocol. https://doi.org/10.17605/OSF.IO/YDC68 ( Parr, 2022). This project contains the following extended data: Search strategy Data extraction form Open Science Framework: PRISMA-P checklist for ‘Public health intelligence challenges for local public health authorities responding to disease outbreaks: a mixed-methods systematic review protocol’ https://doi.org/10.17605/OSF.IO/YDC68 ( Parr, 2022). Data are available under the terms of the Creative Commons Zero "No rights reserved" data waiver (CC0 1.0 Public domain dedication).
