# Supplementary material for: Public health intelligence challenges for local public health authorities responding to disease outbreaks: a mixed-methods systematic review protocol
Source: NIHR Open Res. 2023 Jul 19;2:56. Originally published 2022 Nov 16. [Version 2] doi: 10.3310/nihropenres.13307.2 (PMC10593317; doi:10.3310/nihropenres.13307.2)
Supplement: Peer review history

# Reviewer report: Reviewer response for version 2

**DOI:** 10.3310/nihropenres.14597.r29953

**Published:** 2023-08-02

**Authors:** Ritthideach Yorsaeng

**Affiliations:** 1 Chulalongkorn University, Bangkok, Bangkok, Thailand

After amendments from the first version, this version was clearer than the previous one. The detail and methodology in the amended protocol are appropriate for the systematic review.

 Data sources and search.

- One of the important databases used in medical science and included in many systematic-review is the Web of Science (Clarivate). Why does this protocol not include the Web of Science in the search strategy?

Some of the literature you will miss may be indexed only in this database.

- Other databases that report the potential emerging threat are Bioinformatics databases such as GenBank or GISAID. However, the bioinformatics databases may no suitable for practice without interpretation by expertise, but the information from these databases is fast and reliable.

Is the study design appropriate for the research question?

Yes

Is the rationale for, and objectives of, the study clearly described?

Yes

Are sufficient details of the methods provided to allow replication by others?

Yes

Are the datasets clearly presented in a useable and accessible format?

Partly

Reviewer Expertise:

Clinical trials, Vaccine trials, Immunology, Epidemiology, Public Health

I confirm that I have read this submission and believe that I have an appropriate level of expertise to confirm that it is of an acceptable scientific standard.

# Reviewer report: Reviewer response for version 2

**DOI:** 10.3310/nihropenres.14597.r29940

**Published:** 2023-07-26

**Authors:** Gabriela Mustata Wilson, Ana Aleksandric

**Affiliations:** 1 Multi-Interprofessional Center for Health Informatics (MICHI), The University of Texas at Arlington, Arlington, TX, USA

No further comments to make.

Is the study design appropriate for the research question?

Partly

Is the rationale for, and objectives of, the study clearly described?

Partly

Are sufficient details of the methods provided to allow replication by others?

Partly

Are the datasets clearly presented in a useable and accessible format?

Partly

Reviewer Expertise:

health informatics, public health informatics, computer science

We confirm that we have read this submission and believe that we have an appropriate level of expertise to confirm that it is of an acceptable scientific standard.

# Reviewer report: Reviewer response for version 1

**DOI:** 10.3310/nihropenres.14432.r29143

**Published:** 2023-04-11

**Authors:** Kay Cooper

**Affiliations:** 1 School of Health Sciences, Robert Gordon University, Aberdeen, UK

- This protocol details the review questions and proposed methodology for a mixed methods systematic review investigating public health intelligence challenges for local public health authorities responding to disease outbreaks.
- The review questions seem appropriate for a mixed methods review.
- The search strategy (extended data) is comprehensive.
- Inclusion criteria are clearly detailed.
- The start date for searching (2019) needs justification however. The authors state that the review is not solely focused on COVID-19. It is therefore not clear why searching will only start from the time of the COVID-19 outbreak. Could there not be relevant earlier literature relating to other disease outbreaks (e.g., SARS and others), especially to address Review Question 2 about how local public health intelligence responses evolve over time?
- Study selection mostly follows standard systematic review methodology. However, there is an assumption that any disagreements at title/abstract screening stage will be resolved through discussion, with no mention of a third reviewer where required.
- Data extraction and synthesis/integration also lack clarity. The data extraction tool (extended data) includes 'textual description of quantitative data' and 'thematic analysis'. The former is congruent with the JBI integrated synthesis approach that the authors state they are taking; however the additional step of thematic analysis is not. 
- Quality assessment could be clearer. The MMAT tool is stated 'or a tool suitable for their design'. Given there are so many critical appraisal tools to select from, it would be good for the authors to indicate which tools, or which suite/database they will be selected from.

Is the study design appropriate for the research question?

Yes

Is the rationale for, and objectives of, the study clearly described?

Yes

Are sufficient details of the methods provided to allow replication by others?

No

Are the datasets clearly presented in a useable and accessible format?

Not applicable

Reviewer Expertise:

Applied health research; Mixed methods research; Systematic review methodology

I confirm that I have read this submission and believe that I have an appropriate level of expertise to confirm that it is of an acceptable scientific standard, however I have significant reservations, as outlined above.

# Reviewer report: Reviewer response for version 1

**DOI:** 10.3310/nihropenres.14432.r28948

**Published:** 2023-03-21

**Authors:** Lucylynn Lizarondo

**Affiliations:** 1 JBI, The University of Adelaide, Adelaide, SA, Australia

I think the answers to the questions they posed are quite established in the literature so I'm not sure of the originality of the proposed study. In terms of the design, a mixed methods review would be appropriate however, I am unclear with some of the methods indicated in the protocol. The authors proposed a convergent integrated approach, and in this approach, data transformation is essential to be able to integrate the different types of data. They mentioned synthesis occurring at the data level but also indicated thematic analysis in the data synthesis section---this is unclear. I'm also not convinced that the search should only be limited to those published from Jan 2019, which obviously would capture papers relevant to COVID-19 but not anything else. And this would seem too limited given that the review is not just about COVID-19 but rather disease outbreaks in general.

Is the study design appropriate for the research question?

Yes

Is the rationale for, and objectives of, the study clearly described?

Yes

Are sufficient details of the methods provided to allow replication by others?

Partly

Are the datasets clearly presented in a useable and accessible format?

Not applicable

Reviewer Expertise:

health services, evidence synthesis and implementation methodologies

I confirm that I have read this submission and believe that I have an appropriate level of expertise to confirm that it is of an acceptable scientific standard, however I have significant reservations, as outlined above.

# Reviewer report: Reviewer response for version 1

**DOI:** 10.3310/nihropenres.14432.r28996

**Published:** 2023-02-15

**Authors:** Gabriela Mustata Wilson, Ana Aleksandric

**Affiliations:** 1 Multi-Interprofessional Center for Health Informatics (MICHI), The University of Texas at Arlington, Arlington, TX, USA

The proposal aims to provide a literature review protocol on the public health intelligence challenges relevant to disease outbreaks. This is a pertinent topic, and the systematic review methodology is accurate and easy to follow.

 However, the following items should be included in this analysis:

- The study will capture all the works published after 2019, before, during, and following the COVID-19 pandemic. However, I believe that the public health intelligence activities that were “forced” to emerge rapidly in 2020 would be interesting to compare with the pre-pandemic public health intelligence activities. In more detail, I agree that the research question addressing how public health intelligence responses evolved (RQ2) is very relevant, but the aspect that should also be included is how COVID-19 affected previously existing intelligence responses and how they improved intending to combat the global pandemic, without only focusing on intelligence activities have been undertaken by subnational public health authorities (RQ1).
- Another relevant addition, besides listing available responses, their challenges, gaps, and potential future work might be sorting intelligence responses by their effectiveness or how well they helped resolve disease outbreaks (measuring how good they were in solving the bigger problem). This can give researchers more ideas about which responses to focus on in future work and what kind of responses are preferable compared to the others.
- Does this methodology apply only to disease outbreaks? There might be similar challenges that public health faces that are worth exploring.
- The proposal mentions that the search will be done on articles from 2019 and forward. It might be worth exploring a few years back when social media became popular worldwide, as it could significantly affect public health intelligence tools (i.e., misinformation affecting public health interventions' effectiveness).

However, even though this is a relevant topic, it is unclear how this research will contribute to responding better to public health emergencies. There is a general awareness of the barriers faced regarding data collection, analysis, and information dissemination regarding public health interventions. Even though putting together all the recent intelligence responses and their relevance in one place is valuable, the research questions do not bring any novelty.

Is the study design appropriate for the research question?

Partly

Is the rationale for, and objectives of, the study clearly described?

Partly

Are sufficient details of the methods provided to allow replication by others?

Partly

Are the datasets clearly presented in a useable and accessible format?

Partly

Reviewer Expertise:

health informatics, public health informatics, computer science

We confirm that we have read this submission and believe that we have an appropriate level of expertise to confirm that it is of an acceptable scientific standard, however we have significant reservations, as outlined above.
